# The principal PINK1 and Parkin cellular events triggered in response to dissipation of mitochondrial membrane potential occur in primary neurons

**DOI:** 10.1111/gtc.12066

**Published:** 2013-06-10

**Authors:** Fumika Koyano, Kei Okatsu, Shinsuke Ishigaki, Yusuke Fujioka, Mayumi Kimura, Gen Sobue, Keiji Tanaka, Noriyuki Matsuda

**Affiliations:** 1 Laboratory of Protein Metabolism, Tokyo Metropolitan Institute of Medical ScienceSetagaya-ku, Tokyo, 156-8506, Japan; 2 Department of Medical Genome Sciences, Graduate School of Frontier Sciences, The University of TokyoKashiwa, Chiba, 277-8561, Japan; 3 Department of Neurology, Nagoya University Graduate School of MedicineNagoya, 466-8550, Japan

## Abstract

*PINK1* and *PARKIN* are causal genes for hereditary Parkinsonism. Recent studies have shown that PINK1 and Parkin play a pivotal role in the quality control of mitochondria, and dysfunction of either protein likely results in the accumulation of low-quality mitochondria that triggers early-onset familial Parkinsonism. As neurons are destined to degenerate in PINK1/Parkin-associated Parkinsonism, it is imperative to investigate the function of PINK1 and Parkin in neurons. However, most studies investigating PINK1/Parkin have used non-neuronal cell lines. Here we show that the principal PINK1 and Parkin cellular events that have been documented in non-neuronal lines in response to mitochondrial damage also occur in primary neurons. We found that dissipation of the mitochondrial membrane potential triggers phosphorylation of both PINK1 and Parkin and that, in response, Parkin translocates to depolarized mitochondria. Furthermore, Parkin's E3 activity is re-established concomitant with ubiquitin–ester formation at Cys431 of Parkin. As a result, mitochondrial substrates in neurons become ubiquitylated. These results underscore the relevance of the PINK1/Parkin-mediated mitochondrial quality control pathway in primary neurons and shed further light on the underlying mechanisms of the PINK1 and Parkin pathogenic mutations that predispose Parkinsonism *in vivo*.

## Introduction

Mitochondrial homeostasis plays a pivotal role in the maintenance of normal healthy cells, in particular postmitotic cells such as neurons. Mitochondria are constitutively injured by endogenous and exogenous stresses, such as reactive oxygen species (ROS) and mitochondrial DNA (mtDNA) mutations. Defective mitochondria, if left unchecked, become an aberrant source of oxidative stress due to the generation of excessive ROS and compromise healthy mitochondria through intermitochondrial reciprocity via fusion and fission. Thus, to maintain the integrity and quality of mitochondria, cells establish a mitochondrial quality control system via the selective elimination of impaired mitochondrion (Ashrafi & Schwarz 1).

Parkinson's disease (PD) is one of the most pervasive neurodegenerative diseases. Although the cause of sporadic PD is likely complex, numerous evidences link mitochondrial dysfunction to its pathogenesis. A moderate deficit in mitochondrial activity after exposure to pesticides such as rotenone (a mitochondrial complex I inhibitor) and paraquat (an oxidative stressor) predisposes to PD (Tanner *et al*. 39), and mutations/deletions of mtDNA in patients with PD have repeatedly been reported (Schapira 35). *PINK1* (*P**TEN-**in**duced putative* *k**inase 1*) and *PARKIN* are causal genes for hereditary (i.e. autosomal recessive) early-onset Parkinsonism (Kitada *et al*. 17; Valente *et al*. 40). Although the phenotype of the hereditary early-onset Parkinsonism is not the same as sporadic PD completely, they share a major clinical feature (Imaizumi *et al*. 12). Newly emergent evidences have shown that PINK1 and Parkin play a pivotal role in the quality control of mitochondria, and dysfunction of either likely results in the accumulation of low-quality mitochondria thereby triggering early-onset familial Parkinsonism (Corti *et al*. 6; Exner *et al*. 8). According to the most recently proposed model, PINK1 selectively localizes to low-quality mitochondria by escaping mitochondrial membrane potential (ΔΨm)-dependent degradation and subsequently undergoes autophosphorylation-dependent activation (Jin *et al*. 13; Okatsu *et al*. 30). Activated PINK1 then recruits the latent form of Parkin from the cytosol to the same low-quality mitochondria (Matsuda *et al*. 24; Narendra *et al*. 27; Vives-Bauza *et al*. 42; Okatsu *et al*. 30). Concomitantly, Parkin is phosphorylated at Ser65 in a PINK1-dependent manner (Kondapalli *et al*. 18; Shiba-Fukushima *et al*. 36), and the ubiquitin ligase (E3) activity of Parkin is activated (Matsuda *et al*. 24). Although the molecular mechanism underlying how a decrease in ΔΨm activates Parkin has yet to be completely elucidated, suppression of the autoinhibitory mechanism (Chaugule *et al*. 5) and ubiquitin–thioester formation at Cys431 of Parkin (Lazarou *et al*. 20) (M.I., K.T., and N.M., unpublished data) is thought to be a critical step for up-regulating the E3 activity of Parkin. Once activated, Parkin ubiquitylates outer mitochondrial membrane substrates such as hexokinase I (HKI), MitoNEET/CISD1, mitofusin (Mfn), miro and voltage-dependent anion channel (VDAC) 1 (Gegg *et al*. 9; Geisler *et al*. 10; Tanaka *et al*. 38; Ziviani *et al*. 46; Chan *et al*. 4; Wang *et al*. 43; Yoshii *et al*. 45; Liu *et al*. 22; Okatsu *et al*. 29; Sarraf *et al*. 34; and references therein). As a consequence, damaged mitochondria become quarantined through decreased mitochondrial fusion, separated from the destination (e.g. presynaptic terminal) by a pause in kinesin-dependent anterograde trafficking and/or degraded via autophagy.

The cascading reactions underlying transduction of the PINK1 and Parkin ‘mitochondrial damage’ signal remain a topic of vigorous research. As described above, critical elements of this signal have been recently elucidated; however, several caveats to the current findings are worth highlighting. The most glaring shortcoming is that neuronal studies of PINK1 and Parkin have been limited with almost all aspects of the PINK1/Parkin pathway showed using non-neuronal cell types (e.g. HeLa cells, HEK cells and MEFs). Moreover, a report by Sterky *et al*. (37) seriously undermined the relevance of mitochondrial quality control mediated by PINK1/Parkin in neurons. To address these issues, we examined whether the PINK1/Parkin pathway reported in non-neuronal cells is also observed in primary neurons. Here we show for the first time using mouse primary neurons that both PINK1 and Parkin are phosphorylated after dissipation of ΔΨm and that the E3 activity of Parkin is up-regulated after ubiquitin–ester formation.

## Results

### PINK1 and Parkin are phosphorylated on dissipation of ΔΨm in mouse primary neurons

**Figure 1:**
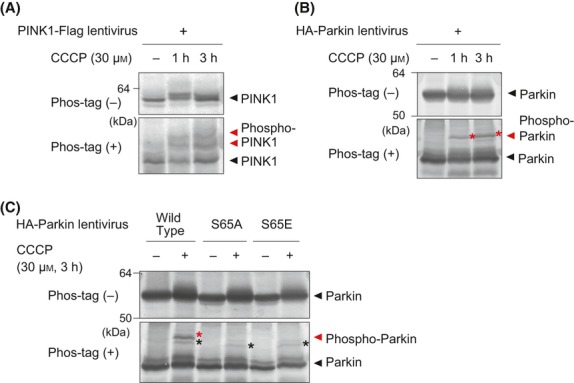
PINK1 and Parkin are phosphorylated after a decrease in ΔΨm in mouse primary neurons. Neurons were infected with lentivirus encoding PINK1-Flag (A), wild-type HA-Parkin (B) or HA-Parkin with either the S65A or S65E mutation (C). Cells were treated with the mitochondrial uncoupler CCCP (30 μm) for 1–3 h and subjected to SDS-PAGE in the absence or presence of 50 μm phos-tag. Note that mobility does not reflect the molecular weight of proteins in phos-tag PAGE (Kinoshita *et al*. 15), and thus, molecular weight markers are not shown in the bottom gels. The red and black asterisks in (C) indicate phosphorylation of Parkin at Ser65 and an additional minor phosphorylation site, respectively.

The most upstream event during PINK1/Parkin-mediated quality control of mitochondria is the discrimination of damaged mitochondria from their healthy counterparts by PINK1 via quantitative and qualitative regulation. Specifically, PINK1 accumulates after a decrease in ΔΨm by escaping from the ΔΨm-dependent degradation pathway. Autophosphorylation of the accumulated PINK1 promotes the efficient retrieval and co-localization of Parkin to damaged mitochondria (Matsuda *et al*. 24; Narendra *et al*. 27; Okatsu *et al*. 30). We first investigated whether PINK1 accumulates and undergoes phosphorylation in response to a decrease in ΔΨm in mouse primary neurons similar to that described in non-neuronal cells. We first tried to detect the endogenous mouse PINK1; however, the currently available anti-PINK1 antibodies were unable to differentiate between *PINK1*^+/+^ and *PINK1*^−/−^ MEFs even after CCCP treatment (M.I. and N.M., unpublished data). We thus used exogenous Flag-tagged human PINK1. At 3 days after dissection, primary neurons were infected with lentivirus encoding PINK1-Flag. Primary neurons expressing PINK1-Flag were then treated with 30 μM carbonyl cyanide *m*-chlorophenylhydrazone (CCCP), which depolarizes mitochondria by increasing membrane permeability to H^+^. The exogenous PINK1 was detected as a doublet in immunoblots of conventional handmade gels (Fig. 1A, upper panel). This higher molecular weight band appeared within 1 h of CCCP treatment and persisted for 3 h. To demonstrate the phosphorylation of PINK1 directly, we carried out a phosphate-affinity SDS-PAGE using polyacrylamide gels conjugated with a 1,3-bis (bis (pyridine-2-ylmethyl) amino) propan-2-oato diMn (II) complex (referred hereafter as phos-tag). Phos-tag can capture phosphomonoester dianions (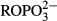), and thus, acrylamide-dependant phos-tag specifically retards the migration of phosphorylated proteins, which are visualized as slower-migrating bands compared with the corresponding nonphosphorylated proteins (Kinoshita *et al*. 16). Phos-tag PAGE demonstrated the phosphorylation of PINK1 in response to ΔΨm dissipation (Fig. 1A, lower panel) concomitantly with doublet formation in normal gels (upper panel).

Previously, several groups reported that Parkin was also phosphorylated at Ser65 on dissipation of ΔΨm in cultured cells (Kondapalli *et al*. 18; Shiba-Fukushima *et al*. 36). To examine whether phosphorylation of Parkin also occurs in neurons, HA-Parkin was exogenously introduced into mouse primary neurons by lentivirus, and the cells were treated with 30 μm CCCP for 1–3 h. Phos-tag PAGE confirmed phosphorylation of Parkin within 1 h of treatment with the phosphorylation signal increasing in intensity over time (Fig. 1B, lower panel). We next checked whether Ser65 is the phosphorylation site used in Parkin. HA-Parkin containing either S65A or S65E mutation was introduced into *PARKIN*^−/−^ mouse primary neurons, which were used to prevent confounding effects from endogenous Parkin. In both mutant lines, the more intense slower-migrating band identified as phosphorylated Parkin in phos-tag PAGE was absent (Fig. 1C, a red asterisk), suggesting that Ser65 is the genuine Parkin phosphorylation site in mouse primary neurons. The presence of a less intense, slightly faster-migrating signal in response to ΔΨm dissipation, even in the S65A/E mutant lines, suggests the presence of a second minor phosphorylation site in Parkin (black asterisks in Fig. 1C).

### Latent E3 activity of Parkin is up-regulated on a decrease in ΔΨm in neurons

**Figure 2:**
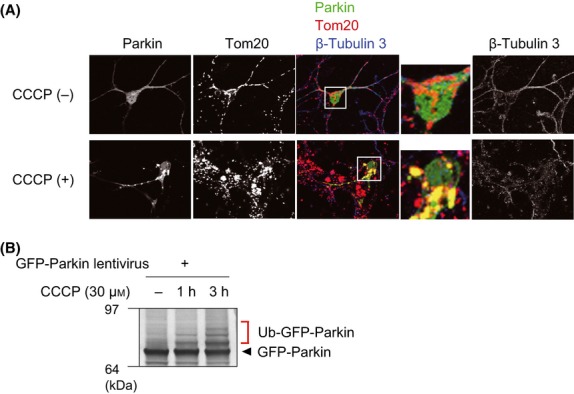
Parkin is recruited to depolarized mitochondria and is activated in neurons. (A) Mouse primary neurons were infected with lentivirus encoding GFP-Parkin and then subjected to CCCP treatment (30 μm) for 3 h. Neurons were immunostained with the indicated antibodies. Insets (white boxes) in the Parkin-, Tom20- and β-tubulin 3-co-immunostained images have been enlarged to better show co-localization. (B) The E3 activity of Parkin was monitored using autoubiquitylation of GFP-Parkin as an indicator. As reported previously (Matsuda *et al*. 24), Parkin ubiquitylates a pseudosubstrate (N-terminally fused GFP) only when the mitochondrial membrane potential decreases. Ub, ubiquitin.

Parkin is selectively recruited to dysfunctional mitochondria with low membrane potential in mammalian cell lines (Narendra *et al*. 25). Moreover, we previously demonstrated that the E3 function of Parkin in cultured cells (e.g. HeLa cells and MEFs) is activated on dissipation of ΔΨm (Matsuda *et al*. 24). Parkin translocation onto neuronal depolarized mitochondria, however, is controversial. Sterky *et al*. (37) and Van Laar *et al*. (41) reported that Parkin failed to localize on depolarized mitochondria after CCCP treatment or by the loss of mitochondrial transcription factor A (TFAM), whereas Cai *et al*. (2) and Joselin *et al*. (14) reported that Parkin relocates to depolarized mitochondria in primary neurons. We thus first examined whether Parkin is recruited to mouse primary neuron mitochondria after CCCP treatment. Neurons were infected with lentivirus encoding GFP-Parkin, and the subcellular localization of Parkin was examined in conjunction with immunofluorescence staining of Tom20 (a mitochondrial outer membrane marker) and β-tubulin isotype 3 (a neuron-specific marker). Under these experimental conditions, Parkin dispersed throughout the cytoplasm under steady-state conditions, whereas Parkin co-localized with depolarized mitochondria (*t* = ∼3 h) after treatment with CCCP (Fig. 2A). We next assessed the E3 activity of Parkin in primary neurons. GFP-Parkin can be ubiquitylated as a pseudosubstrate by Parkin *in cell* (Matsuda *et al*. 23, 24). As a consequence, autoubiquitylation of GFP-Parkin can be used as an indicator of Parkin E3 activity. As shown in Fig. 2B, autoubiquitylation of GFP-Parkin clearly increased after a decrease in ΔΨm, suggesting that latent E3 activity of Parkin is activated on mitochondrial damage in neurons as previously reported in cultured cell lines (e.g. HeLa cells).

### Pathogenic mutations impair the E3 activity of Parkin and inhibit mitochondrial localization

**Figure 3:**
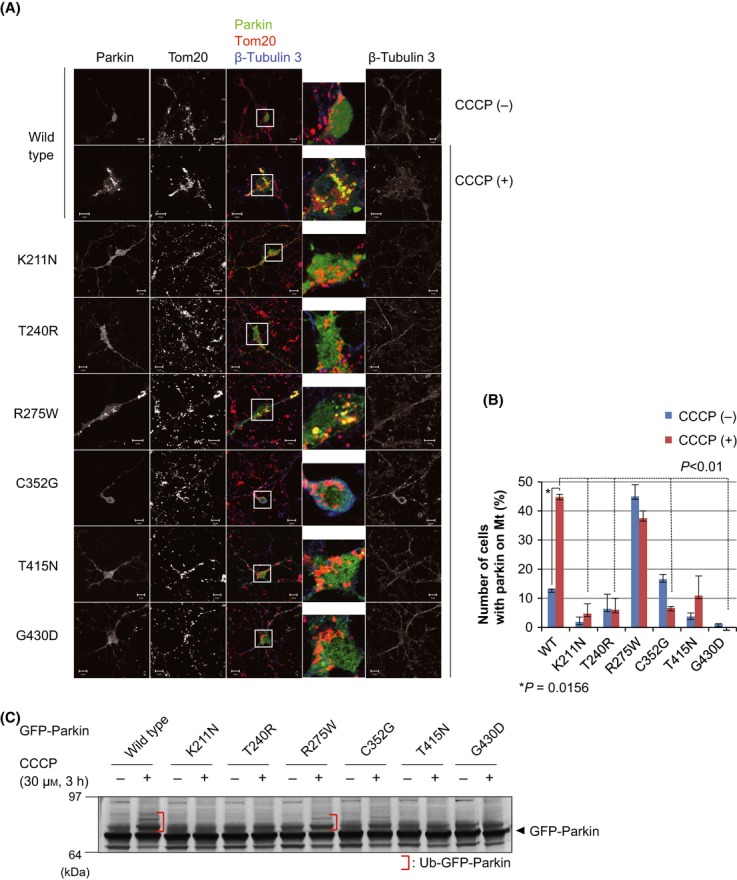
Disease-relevant Parkin mutations impair mitochondrial localization and E3 activity after CCCP treatment. (A) The subcellular localization of GFP-Parkin with pathogenic mutations in the isolated neurons from *PARKIN* knockout (*PARKIN*^−/−^) mice. Primary neurons were infected with lentivirus encoding GFP-Parkin containing various disease-relevant mutations and then treated with CCCP (30 μm) for 3 h, followed by immunocytochemistry, as in Fig. 2A. (B) The number of neurons with GFP-Parkin-positive mitochondria was counted. Error bars represent the mean ± SD values of two experiments. Statistical significance was calculated using analysis of variance with a Student's *t*-test. (C) The E3 activity of Parkin with disease-relevant Parkin mutations. *PARKIN*^−/−^ primary neurons expressing pathogenic GFP-Parkin were treated with CCCP for 3 h and subjected to immunoblotting with an anti-Parkin antibody.

To further verify that the events shown in Fig. 2 are aetiologically important, we selected six pathogenic mutants of Parkin (K211N, T240R, R275W, C352G, T415N and G430D) and examined their subcellular localization and E3 activity. To eliminate the effect of endogenous Parkin, we used primary neurons derived from *PARKIN*^−/−^ mice in these experiments. The six GFP-Parkin mutants were serially introduced into *PARKIN*^−/−^ primary neurons using a lentivirus and assayed for their subcellular localization after CCCP treatment. Parkin mitochondrial localization was compromised by the K211N (mutation in RING0 domain), T240R (in RING1 domain), C352G (in IBR domain), T415N and G430D (both in RING2 domain) mutations (Fig. 3A). The defects seen with the K211N, T240R, C352G and G430D mutants (Fig. 3B), in contrast to T415N *(P *> 0.01), were statistically significant (*P *< 0.01). The R275W mutation had no effect on mitochondrial localization after CCCP treatment. The E3 activity of the mutants was also assessed. The K211N, T240R, C352G, T415N and G430D mutations exhibited deficient autoubiquitylation activity in *PARKIN*^−/−^ primary neurons (Fig. 3C). The R275W mutant had weak but reproducible autoubiquitylation activity after CCCP treatment. Because this mutant showed partial mitochondrial localization after CCCP treatment even in HeLa cells (Okatsu *et al*. 31; Lazarou *et al*. 20), it is not surprising that the R275W mutant localizes to neuronal depolarized mitochondria and possesses weak E3 activity. Unexpectedly, the R275W mutant also localized to mitochondria even in the absence of CCCP treatment. Although the significance of R275W localization to healthy mitochondria is unknown, we propose that the R275W mutation maintains Parkin in an inactive state (as suggested by Fig. 3C) because functional, phosphorylated PINK1 has not been reported in normal mitochondria. In most of the pathogenic Parkin mutants, translocation to damaged mitochondria and conversion to the active form were compromised after a decrease in ΔΨm (Fig. 3), suggesting the aetiological importance of these events in neurons.

### Parkin forms an ubiquitin–thioester intermediate in mouse primary neurons

**Figure 4:**
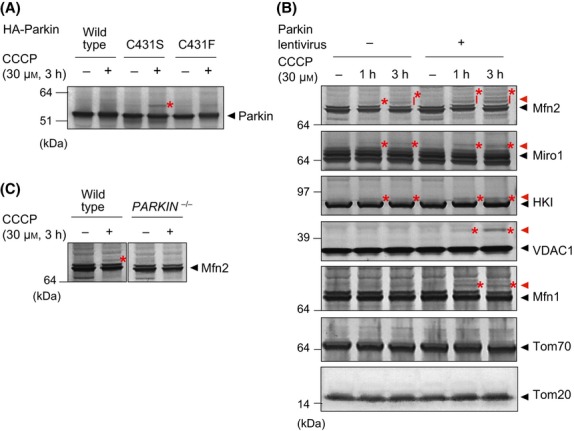
Several outer membrane mitochondrial proteins underwent Parkin-dependent ubiquitylation after a decrease in the membrane potential. (A) Ubiquitin–oxyester formation on Parkin (shown by the red asterisk) was specifically observed in the Parkin C431S mutant after CCCP treatment in primary neurons. This modification was not observed in wild-type Parkin or the C431F mutant. (B) Intact primary neurons, or primary neurons infected with lentivirus encoding Parkin, were treated with CCCP and then immunoblotted to detect endogenous Mfn2, Miro1, HKI, VDAC1, Mfn1, Tom70 and Tom20. The red arrowheads and asterisks indicate ubiquitylated proteins. (C) Ubiquitylation of Mfn2 after mitochondrial depolarization (shown by the red asterisk) is prevented by *PARKIN* knockout in primary neurons.

Klevit's group recently reported that Cys357 in the RING2 domain of RBR-type E3 HHARI is an active catalytic residue and forms an ubiquitin–thioester intermediate during ubiquitin ligation (Wenzel *et al*. 44). Parkin is also a RBR-type E3 with Parkin Cys431 equivalent to HHARI Cys357. We and a number of groups recently independently showed that a Parkin C431S mutant forms a stable ubiquitin–oxyester on CCCP treatment in non-neuronal cell lines, suggesting the formation of an ubiquitin–thioester intermediate (Lazarou *et al*. 20) (M.I., K.T., and N.M., unpublished data). To examine whether Parkin forms an ubiquitin–ester intermediate in neurons as well, we again used a lentivirus to express HA-Parkin with the C431S mutation, which converts an unstable ubiquitin–thioester bond to a stable ubiquitin–oxyester bond. The HA-Parkin C431S mutant specifically exhibited an upper-shifted band equivalent to an ubiquitin–adduct after CCCP treatment (Fig. 4A, lane 4). This modification was not observed in wild-type HA-Parkin (lane 2) and was absent when an ester-deficient pathogenic mutation, C431F, was used (lane 6), suggesting ubiquitin–oxyester formation of Parkin when neurons are treated with CCCP.

Finally, we examined whether specific mitochondrial substrates undergo Parkin-mediated ubiquitylation in primary neurons. The ubiquitylation of Mfn1/2, Miro1, Tom20, Tom70, VDAC1 and hexokinase I (HKI) (Gegg *et al*. 9; Geisler *et al*. 10; Poole *et al*. 32; Tanaka *et al*. 38; Ziviani *et al*. 46; Chan *et al*. 4; Glauser *et al*. 11; Rakovic *et al*. 33; Wang *et al*. 43; Yoshii *et al*. 45; Liu *et al*. 22; Narendra *et al*. 26; Okatsu *et al*. 29; Sarraf *et al*. 34) was evaluated by Western blotting. In initial experiments using primary neurons, detection of the ubiquitylated mitochondrial substrates (e.g. Mfn) was minimal (F.K. and N.M., unpublished data). We thus changed various experimental conditions and determined that ubiquitylation of mitochondrial substrates became detectable when the primary neurons were cultured in media free of insulin, transferrin and selenium (described in detail in Experimental procedures). Although these compounds are routinely added to the neuronal medium as antioxidants to reduce excessive ROS in primary neurons, their exclusion facilitated the detection of ubiquitylated mitochondrial substrates (see Discussion). Higher molecular mass populations of endogenous Mfn1/2, Miro1, HKI and VDAC1 were observed after CCCP treatment, and this was particularly evident in neurons expressing exogenous Parkin (Fig. 4B). The modification resulted in a 6- to 7-kDa increase in the molecular weight, strongly suggestive of ubiquitylation by Parkin, as has been reported previously in non-neuronal cells. Moreover, in *PARKIN*^−/−^ primary neurons, the modification of Mfn2 was not observed after CCCP treatment (Fig. 4C, compare lane 2 with lane 4), confirming that Mfn undergoes Parkin-dependent ubiquitylation in response to a decrease in ΔΨm.

## Discussion

Recently, several reports on PINK1 and Parkin have contributed significantly to our understanding of their *in vivo* functionality. Most of these studies, however, have used non-neuronal cultured cell lines such as HeLa and HEK cells. To elucidate the physiological role of PINK1 and Parkin underlying the onset of hereditary Parkinsonism, evaluation of their role under more physiological conditions such as in neurons is imperative. We therefore sought to establish a mouse primary neuron experimental system to address this issue.

In our initial experiments, ubiquitylation of mitochondrial substrates (e.g. Mfn) in primary neurons after CCCP treatment was below the threshold of detection. We thus changed various experimental conditions including the composition and inclusion of supplementary factors to the culture medium. We determined that detection of ubiquitylation was improved when the primary neurons were cultured in media free of insulin, transferrin and selenium. Transferrin plays a role in the reduction of toxic oxygen radicals, although selenium in the medium accelerates the antioxidant activity of glutathione peroxidase. Thus, a weak oxidative stress to neuronal mitochondria seems to accelerate the ubiquitylation of mitochondrial substrates by Parkin. Because oxidative stress is assumed to be a primary stress for neuronal mitochondria *in vivo* (Navarro *et al*. 28), this mechanism is thought to be critical for efficiently rescuing abnormal mitochondria under physiological conditions. Moreover, it has also been reported that oxidative stress helps Parkin exert mitochondrial quality control in neurons (Joselin *et al*. 14). Although the molecular mechanism underlying how weak oxidative stress accelerates Parkin-catalyzed ubiquitylation remains obscure, we speculate that deubiquitylase activity in neuronal mitochondria conceals the ubiquitylation signal under steady-state conditions. This activity is down-regulated by oxidative stress (Cotto-Rios *et al*. 7; Kulathu *et al*. 19; Lee *et al*. 21). Intriguingly, the Mfn2 ubiquitylation-derived signal in primary neurons remained fainter than that observed in cultured cells even using antioxidant-free media (Gegg *et al*. 9; Tanaka *et al*. 38). In this respect, we speculate that differences in the intracellular metabolic pathways between primary neurons and cultured cell lines affect ubiquitylation of mitochondrial substrates. Van Laar *et al*. (41) reported that Parkin does not localize to depolarized mitochondria in cells forced to dependence on mitochondrial respiration, for example, galactose-cultured HeLa cells. If so, ubiquitylation of mitochondrial substrates by Parkin would be less efficient because neurons have a higher dependency for mitochondrial respiration than other cultured cells.

In contrast to the ubiquitylation of mitochondrial substrates, we obtained clearer results concerning the other principal PINK1 and Parkin events after dissipation of ΔΨm, that is, phosphorylation of PINK1 and Parkin (Fig. 1), translocation of Parkin to the depolarized mitochondria and re-establishment of Parkin's E3 activity toward pseudosubstrates concomitant with ubiquitin–ester formation at Cys431 (Figs 4). These data are consistent with what has been reported using non-neuronal cultured cells. In neurons, though, the translocation of Parkin onto damaged mitochondria is controversial. Initial efforts failed to detect Parkin localization to damaged neuronal mitochondria (Sterky *et al*. 37; Van Laar *et al*. 41). Subsequent studies, however, by two different groups in addition to us have successfully demonstrated the translocation event [(Cai *et al*. 2; Joselin *et al*. 14) and this work]. We suggest that methodological differences likely account for the seemingly conflicting observations. The study by Sterky *et al*. used adeno-associated virus encoding mCherry-Parkin that was delivered by stereotactic injections to midbrain dopaminergic neurons of Tfam-loss mice (MitoPark mice; genotype *Tfam*^loxP/loxP^; DAT-*cre*; ROSA26^+/lox-Stop-lox-mito-YFP^) (Sterky *et al*. 37), although Van Laar *et al*. (41) used Lipofectamine 2000 to transfect wild-type rat primary cortical neurons with human Parkin. In contrast, we used primary neurons derived from *PARKIN*^−/−^ mice infected with a lentivirus encoding GFP-Parkin to examine translocation of Parkin to damaged mitochondria. It is possible that the respective transfection efficiencies varied or that the methodological differences affected the neuronal cellular conditions, which may have impaired the behavior of exogenous Parkin. Alternatively, the presence of endogenous neuronal Parkin may account for the discrepancies. During our immunofluorescence experiments, we determined that mitochondrial localization of GFP-Parkin was more robust in *PARKIN*^−/−^ neurons than wild-type (*PARKIN*^+/+^) neurons (F.K. and N.M., unpublished data), suggesting that endogenous Parkin is more efficiently translocated by the cellular machinery to depolarized mitochondria than exogenous Parkin. Intriguingly, both the E3 activity and translocation of Parkin toward depolarized mitochondria were attenuated by disease-relevant Parkin mutations in primary neurons (Fig. 3). These results underscore the relevance of mitochondrial quality control mediated by PINK1/Parkin in neurons and shed light on the mechanism by which pathogenic mutations of PINK1 and Parkin predispose to Parkinsonism *in vivo*.

## Experimental procedures

### Lentivirus

*HA-PARKIN*, *GFP-PARKIN* or *PINK1-Flag* genes were cloned into a lentiviral vector (pLenti-CMV puro DEST, a kind gift from Dr. Eric Campeau at Resverlogix Corp.). Lentivirus was prepared following Campeau's protocols (Campeau *et al*. 3). Briefly, lentiviral particles were produced in HEK293T cells by transfection of the aforementioned lentiviral vectors using Lipofectamine 2000 (Life Technologies). A lentivirus-containing supernatant was collected 48 h after transfection and concentrated to 10× by ultracentrifugation at 37,000 × ***g*** for 2 h.

### Primary neuron culture

Mouse studies were approved by the Animal Care and Use Committee of Tokyo Metropolitan Institute of Medical Science. Mouse fetal brains were taken from C57BL/6 wild-type or *PARKIN*^−/−^ mouse embryos at E15-16. After removing meninges, brain tissue was dissociated into a single-cell suspension using a Sumilon dissociation solution (Sumitomo Bakelite, Japan). Cells were plated at a density of 3–4 × 10^5^ cells/mL on poly-L-lysine (Sigma)-coated dishes with the medium containing 0.33× Sumilon nerve-culture medium (Sumitomo Bakelite), 0.67% FBS (Equitech-bio, USA), 0.67× neurobasal medium, 0.67× B27 supplements, 0.67× Glutamax (above three reagents are from Life Technologies) and 0.67% Pen-Strep. Three days after plating (at day 4), neurons were infected with lentivirus containing *HA-PARKIN, GFP-PARKIN* or *PINK1-Flag*. After 4 h of infection, the virus medium was removed. Neurons were treated with CCCP (30 μm) for 1–3 h at day 7 and then harvested for immunoblotting or subjected to immunocytochemistry.

### Conventional and phos-tag immunoblotting

To detect ubiquitylation and phosphorylation, lysates of mouse primary neurons were collected in TNE-N^+^ buffer [150 mm NaCl, 20 mm Tris–HCl (pH 8.0), 1 mm EDTA and 1% NP-40] in the presence of 10 mm *N*-ethylmaleimide (Wako chemicals) to protect ubiquitylated proteins from deubiquitylase and phosSTOP (Roche) to protect phosphorylated proteins from phosphatase activity. To detect phosphorylated proteins by PAGE, 7.5% polyacrylamide gels containing 50 μM phos-tag acrylamide (Wako chemicals) and 100 μm MnCl_2_ were used. After electrophoresis, phos-tag acrylamide gels were washed with transfer buffer containing 0.01% SDS and 1 mm EDTA for 10 min with gentle shaking and then washed with transfer buffer containing 0.01% SDS without EDTA for 10 min according to the manufacturer's protocol. Proteins were transferred to polyvinylidene difluoride membranes and analyzed by conventional immunoblotting. Image contrast and brightness were adjusted in Photoshop (Adobe).

### Immunocytochemistry

Primary neuron cells were fixed with 4% paraformaldehyde, permeabilized with 50 μg/mL digitonin and stained with primary antibodies described below and with the following secondary antibodies: mouse and rabbit Alexa Fluor 568 and 647 (Life Technologies). Neurons were imaged using a laser scanning microscope (LSM780; Carl Zeiss, Inc.).

### Antibodies

Antibodies used in this study are as follows: anti-Tom20 (FL145; Santa Cruz Biotech.), anti-Parkin (PRK8; Sigma), anti-Tom70 (gift from Dr. Otera), anti-β-Tubulin isotype 3 (SDL.3D10; Sigma), anti-Miro1 (RHOT1; Sigma), anti-Mitofusin2 (ab56889; Abcam), anti-VDAC1 (ab-2; Calbiochem), anti-PINK1 (BC100-494; Novus) and anti-HKI (C35C4; Cell Signaling) antibodies.
